# Validation and measurement invariance of the Langer mindfulness scale: the Turkish version

**DOI:** 10.3389/fpsyg.2024.1474577

**Published:** 2024-11-21

**Authors:** Menekşe Uysal Saraç, Yıldız Yıldırım, Hasan Eşici, Şener Büyüköztürk, Francesco Pagnini, Ellen Langer

**Affiliations:** ^1^Department of Educational Sciences, Çankırı Karatekin University, Çankırı, Türkiye; ^2^Department of Educational Sciences, Aydın Adnan Menderes University, Aydın, Türkiye; ^3^Department of Turkish and Social Sciences Education, Gazi University, Ankara, Türkiye; ^4^Department of Educational Sciences, Hasan Kalyoncu University, Gaziantep, Türkiye; ^5^Department of Psychology, Università Cattolica del Sacro Cuore, Rome, Italy; ^6^Department of Psychology, Harvard University, Cambridge, MA, United States

**Keywords:** Langer mindfulness scale, scale adaptation, validation, measurement invariance, Langerian mindfulness

## Abstract

Novel distinction drawing is an active process that characterizes mindfulness, which has been associated with an open, creative, and probabilistic mental state, as well as the ability to examine information from new perspectives. The literature review revealed a lack of measurement tools for assessing mindfulness from a social and cognitive perspective in Türkiye. In addition, the frequent use of the Langer Mindfulness Scale (LMS) in educational contexts and its adaptation into many languages highlights the need for a Turkish version of the scale. This study aims to validate the Turkish version of the 14-item LMS. The Turkish version’s factorial structure was tested using the results of confirmatory factor analysis (CFA), which confirmed a three-factor structure that included the engagement, novelty-producing, and novelty-seeking subscales. It was found that measurement invariance based on gender was provided by LMS scores. LMS Turkish version showed satisfying psychometric properties in terms of reliability. Additionally, convergent and discriminant validity were examined in this validation study to provide evidence for criterion-based validity. For this purpose, the relationships between Turkish LMS scores and variables such as positive and negative affect, openness to experiences, self-acceptance, self-defined humor, and health were analyzed. The results showed that self-defined humor, positive affect, openness to experience, and self-acceptance were significantly positively correlated with the Turkish LMS scores, while LMS scores exhibited a significant negative relationship with negative affect. These findings suggest that the Turkish version of the LMS, with its three dimensions, shows acceptable psychometric properties for assessing the state of mindfulness. The Turkish version of the LMS is expected to be used in socio-cognitive mindfulness research in the Turkish cultural context.

## 1 Introduction

Mindfulness has its roots in Asian psychology, originating from Buddhist traditions (Gilbert, 1973; Stoops, 2005). One of the earliest definitions was provided by Buddhist teacher Thich Nhat Hanh, a prominent figure in the pursuit of world peace. Hanh (1991) defined mindfulness as the awareness of the present moment, highlighting its significance as the foundation of a happy life. He also used this concept as a meditation technique to bring people back to the present moment, enabling them to become aware of their thoughts, behavior, and emotions (Stoops, 2005).

According to Bishop et al. (2004), in modern psychology, mindfulness is recognized as an awareness-enhancing method that effectively addresses thought processes that contribute to emotional stress and maladaptive behavior. Kabat-Zinn (2003) further defined mindfulness in functional terms, explaining it as the awareness that arises when an individual focuses on the present moment, continually attending to the unfolding of experiences without prejudice.

Furthermore, Langer and Moldoveanu (2000) emphasized the difficulty of defining the concept of mindfulness and attempted to explain it as follows: Mindfulness is the process of drawing novel distinctions. As long as this distinction is novel, its importance or triviality is irrelevant. Mindfulness, enabling the discovery of similarities between seemingly different things and differences in seemingly similar things, represents an open, creative, and probabilistic mental state arising from examining information from fresh perspectives and being sensitive to context (Langer, 1993). Awareness of these differences and similarities actively grounds us in the present moment. It also allows us to be more aware of those moments when we rely on previously established categories and differences. Otherwise, our behavior becomes governed by rules and routines, resulting in a state of unconsciousness. Focusing on authentic differences leads to: (1) heightened environmental sensitivity, (2) openness to new information, (3) creation of new categories for structuring perceptions, and (4) increased awareness of multiple perspectives in problem-solving (Langer and Moldoveanu, 2000).

Mindfulness is a prominent concept in both education and psychology, particularly as unconscious learning frequently occurs in educational settings. Moreover, conventional teaching methods may be used unconsciously. Therefore, creating conscious learning and teaching environments is essential. Many studies can be frequently encountered in the relevant literature on the use of the concept of mindfulness in learning and teaching environments (Davenport and Pagnini, 2016; Sherretz, 2011), in addition to its presence in business environments and the psychological structures it is related to (Junça Silva and Caetano, 2021; Moafian et al., 2017; Pirson et al., 2012). Mindful activities, such as learning, teaching, and working, are expected to exhibit three characteristics: (1) continually creating new categories, (2) openness to novel information, and (3) awareness of different perspectives (Langer, 2016). Research has shown that many measurement tools based on different theories have been developed to measure mindfulness, which stands out in different fields (Baer et al., 2004; Buchheld et al., 2001; Chadwick et al., 2008; Davis et al., 2009; Erus and Deniz, 2018). A literature review on mindfulness scales previously adapted into Turkish is given below.

### 1.1 Literature review

A review of the literature on instruments designed to assess mindfulness reveals the presence of a scale known as the Mindful Attention Awareness Scale (MAAS). This scale was initially developed by Brown and Ryan (2003) and subsequently adapted into Turkish by Özyeşil et al. (2011). The scale was developed to assess individual differences in the frequency of attention states over time and consists of a single factor. Another scale to measure mindfulness is called “The Freiburg Mindfulness Inventory,” which was converted into a shorter form in 2006 (Walach et al., 2006). The researchers stated that the short scale was sensitive to change and could be administered to individuals without previous meditation experience. That unidimensional scale was adapted into Turkish (Karatepe and Yavuz, 2019). Moreover, the “Five Facets Mindfulness Questionnaire (FFMQ)” is another scale developed to measure mindfulness as a five-factor scale developed by Baer et al. (2006) and adapted into Turkish by Kınay (2013). The dimensions of the scale are as follows: (1) non-reactivity to inner experience, (2) observing/noticing/attending to sensations/perceptions/thoughts/feelings, (3) acting with awareness/automatic pilot/concentration/non-distraction, (4) describing/labeling with words, and (5) non-judging of experience. Further, the analysis of the factors has revealed that the Kentucky Inventory of Mindfulness Skills (KIMS) (Baer et al., 2004) and the FFMQ base the structure of mindfulness on a similar definition or theory. Later, a short version of the FFMQ (FFMQ-S) was prepared by Tran et al. (2013) and adapted into Turkish by Ayalp and Hisli-Şahin (2018). In addition, the Toronto Mindfulness Scale (TMS), which also aims to measure mindfulness, was developed by Lau et al. (2006) and adapted into Turkish by Hisli-Şahin and Yeniçeri (2015). The TMS consists of two factors, namely curiosity and decentering. While developing the scale, the researchers wrote the items based on the operational definition of Bishop et al. (2004). Another related scale in the literature is “The Cognitive and Affective Mindfulness Scale (CAMS), developed by Feldman et al. (2007). This scale consists of four factors: attention, present focus, awareness, and acceptance. The researchers aim to provide a scale that adequately represents the four mindfulness components specified in the functional definitions of Bishop et al. (2004) and Kabat-Zinn (2003). Feldman et al. (2007) also presented a 10-item version named “The Cognitive and Affective Mindfulness Scale-Revised (CAMS-R).” This revised version was adapted into Turkish by Çatak (2012).

Moreover, consisting of two factors as independent components, namely present-moment awareness, and acceptance, the “Philadelphia Mindfulness Scale (PHLMS)” was developed by Cardaciotto et al. (2008) and was later adapted into Turkish by Çelik and Onat Kocabıyık (2018). According to Cardaciotto et al. (2008), both PHLMS and KIMS measure the two basic components specified in the definitions proposed by Bishop et al. (2004) and Kabat-Zinn (2003). Another scale, “Adult Mindfulness Scale (AAMS),” was developed by Droutman et al. (2018) and adapted into Turkish by Sarıçam and Çelik (2018). Droutman et al. (2018) stated that they mainly relied on the function-based definition of Bishop et al. (2004) when writing the scale items.

Among the other tools developed to measure mindfulness and adapted into Turkish is the “Mindfulness in Teaching Scale,” developed by Frank et al. (2016) and adapted by Gördesli et al. (2019). In developing this two-factor scale, the researchers benefited from their own expertise while writing the items. They reviewed existing scales such as MAAS and included the items that they thought could reflect the characteristics of mindfulness in the context of teaching. Besides these, the “Mindfulness in Parenting Questionnaire (MIPQ)” was developed by McCaffrey et al. (2017) and subsequently adapted by Gördesli et al. (2018). The items in the first factor of the two-factor scale reflect parenting awareness, non-reactivity in parenting, and goal-focused parenting. In contrast, the other factor reflects present-centered attention, empathic understanding of the child, and acceptance. It can be seen that the structures reflected by the items of the scale are similar to those mentioned previously.

Research has shown that most existing mindfulness scales are rooted in Eastern philosophy, primarily drawing from the definitions established by Bishop et al. (2004) and Kabat-Zinn (2003). However, the LMS distinguishes itself from these scales by being grounded in information processing and creativity theory, thus having a cognitive foundation (Haigh et al., 2011). Furthermore, there are notable differences between Western and Eastern perspectives on mindfulness (Sauer et al., 2013). The theoretical framework underpinning the LMS aligns well with educational contexts, paving the way for studies that explore the application of mindfulness in education (Brown and Langer, 1990; Langer, 1993; Langer, 2014; Langer, 2016; Langer and Moldoveanu, 2000).

Additionally, various adaptations of the LMS have been made across many different cultures. Examples of these adaptations include versions in German (Haller, 2015), Persian (Moafian et al., 2017), Italian (Pagnini et al., 2018), Portuguese (Junça Silva and Caetano, 2021), Japanese (Yang et al., 2023), Brazilian-Portuguese (Fernandes et al., 2023), and French (Cook et al., 2023). This suggests that the LMS is widely utilized to assess mindfulness across diverse cultural contexts.

Given the specific characteristics and relevance of Langer’s approach to mindfulness, it is crucial that the LMS be translated and validated cross-linguistically and cross-culturally so that other researchers and communities might benefit from it, as previously noted by Moafian et al. (2017). Currently, the absence of a mindfulness scale in the research literature in Türkiye that aligns with the Langerian perspective—especially in relation to education—underscores the necessity for adapting the LMS to Turkish.

From this perspective, this study aims to adapt the Langer Mindfulness Scale, originally developed by Langer (2004) with 21 items and four factors: novelty seeking, novelty producing, engagement, and flexibility, and later shortened by Pirson et al. (2012) to 14 items and three factors excluding flexibility, to Turkish culture (i.e., the Langer Mindfulness Scale for Turkish-14 items). In summary, adapting the LMS to Turkish is of considerable importance because it is cognitive-based and used in an educational context. Since a mindfulness scale with these characteristics has not been developed or adopted in Turkish culture, it is thought that the scale will be used by researchers in psychology and education.

## 2 Materials and methods

### 2.1 Scale adaptation process

Upon deciding on the scale to be adapted, the necessary permission was asked for and then granted by Prof. Ellen Langer— the member of the team that developed the scale—to adapt it into Turkish. The steps to be followed in the scale adaptation process were as follows (Hambleton and Patsula, 1999; Hambleton et al., 2005; International Test Commission, 2010):

1. *Selection of qualified translators:* The scale in the source language was translated into the target language. For that purpose, translators who were proficient in both languages, familiar with both cultures, had a good command of the test development process, and had a certain level of knowledge about the measured construct were selected. The translators were composed of five experts: three experts in the field of assessment and evaluation, one expert in English language teaching, and one expert in guidance and psychological counseling.
2. *Translating and adapting the scale:* The forward translation method was employed from among judgmental designs to adapt the scale. Because the review focuses on the scale’s source and target language versions, forward translation designs are the most technically sound (Hambleton and Patsula, 1999). As specified by this method, one or more translators translated the test form from the source language into the target language. The equivalence of these two test versions was then assessed by another group of translators. The advantage of this method is that it focuses directly on the equivalence of the test versions in the source and target languages. Five translators translated the scale items and instructions into the target language.
3. *Reviewing the adapted test and making necessary corrections:* A total of 10 experts were consulted to examine the cultural and linguistic equivalence of the scale items and instructions translated with the forward translation method. Four of them are experts in assessment and evaluation, three in psychological counseling and guidance, two in psychology, and one in Turkish teaching. These experts analyzed the scale for errors that might cause semantic differences between the two language versions. The experts analyzed the translation in terms of the clarity of the sentence and the accuracy of the translation, the difficulty level of the words, and the fluency of the translated text. Regarding the linguistic equivalence of the scale, the experts initially analyzed the scale items for “semantic,” “idiomatic,” “experiential,” and “conceptual” equivalence and then presented their related opinions. The scale was organized according to the feedback received from the experts, and a pilot study form was prepared.
4. *Pre-testing of the adapted test:* The pilot form was organized and administered to adult pre-service teachers studying at the education faculty in different years. During these activities, feedback on the items was received through informal small talk with the students, and the available data were analyzed with the Confirmatory Factor Analysis (CFA). The scale was re-administered after the implementation of adjustments based on feedback and CFA results.
5. *Conducting an appropriate validation study:* The pre-service teachers were again administered the revised scale form. The CFA was performed on the data obtained to examine the construct validity of the scale. In addition, the correlation of the scores obtained from the scale with other variables and measurement invariance were examined.

### 2.2 Participants

First, when the power analysis was performed with the R Shiny semPower 2 package (Moshagen and Bader, 2024), the minimum sample size was determined as 255. For this reason, when some data were excluded from the analysis due to missing data and response patterns (for example, those who marked the same point in every item), the pilot study analyses were conducted on the remaining 400 pre-service teachers. To ensure maximum diversity, the participants were selected from different departments (mathematics, physics, art, biology, English, and so on). Students as participants were in different year levels (1, 2, 3, and 4) at a university in Ankara; 92 (23%) of them were male and 308 (71%) were female.

For validation, the final form of the scale was administered to 490 pre-service teachers studying at the faculties of education of universities in different provinces of Türkiye to examine the construct validity and measurement invariance based on gender. That procedure was carried out through Google Forms. The pre-service teachers studied in different departments (mathematics, Turkish, history, preschool, chemistry, and so on) and different year levels (1, 2, 3, and 4). Of all the participants, 100 (20.4%) were male and 390 (79.6%) were female.

The form was administered via Google Forms to 659 pre-service teachers studying at universities’ faculties of education in different provinces of Türkiye to examine the criterion-based validity. The pre-service teachers studied in different departments (classroom teaching, geography, music, special education, and so on) and different year levels (1, 2, 3 and 4). Those included in the validation process were 141 (21.4%) male and 518 (78.6%) female.

### 2.3 Measurement tools

This study used the LMS developed by Langer (2004) and adapted into a shorter form by Pirson et al. (2012). The scale consists of 14 items and 3 factors and is a 7-point Likert-type questionnaire given as follows: (1= strongly disagree to 7 = strongly agree). The correlations with other variables were analyzed to examine criterion-based validity. The variables and scales used for this purpose are as follows:

#### 2.3.1 Self-reported health

In the final form of the scale, individuals were asked to evaluate their health status between 1 and 10 (1-very unhealthy ➔ 10-very healthy).

#### 2.3.2 Self-defined humor

The participants’ evaluations about self-defined humor were measured with the item: “Other people think that I have a good sense of humor,” which was scaled between 1 and 7 (1 - strongly disagree ➔ and 7 - strongly agree).

#### 2.3.3 Mindful attention and awareness scale (MAAS)

Brown and Ryan (2003) developed MAAS based on an Eastern understanding of mindfulness. Özyeşil et al. (2011) adapted this into Turkish. The scale had one factor and consisted of 15 items. Within the scope of the present study, the fit indices of the scale as a result of CFA were found as follows: *χ*^2^/df = 4.86, RMSEA = 0.077, CFI = 0.979, TLI = 0.967, and SRM*R* = 0.031, all suggesting that the model is an excellent fit. Cronbach’s *α* for the reliability of the measurements was 0.871.

#### 2.3.4 The big five inventory-openness to experience (BFI-openness)

The Big Five Inventory was initially developed by Benet-Martinez and John (1998), subsequently adapted into Türkish and finally reported in the study conducted by Sümer et al. (2005). A total of 10 items in the openness sub-dimension of this inventory were employed in the present study. Based on the CFA results, the fit indices were *χ*^2^/df = 3.73, RMSEA = 0.073, CFI = 0.935, TLI = 0.908, and SRM*R* = 0.043, all indicating that the model is an excellent fit. Cronbach’s *α* for the reliability of the measurements was 0.794.

#### 2.3.5 Positive and negative affect scale (PANAS)

The Positive and Negative Affect Scale (PANAS) was developed by Watson et al. (1988) and subsequently adapted into Turkish by Gençöz (2000). It comprises 20 items, measuring 10 positive and 10 negative emotions. The CFA results for the positive affect subscale (PANAS-PA) were *χ*^2^/df = 4.189, RMSEA = 0.070, CFI = 0.984, TLI = 0.977, and SRM*R* = 0.024. Cronbach’s α taken for the reliability of positive affect measurements was 0.873. For the dimension of negative affect (PANAS-NA), on the other hand, the fit indices were found as *χ*^2^/df = 5.04, RMSEA = 0.078, CFI = 0.983, TLI = 0.972, and SRM*R* = 0.038. Based on this, the model can be considered an excellent fit. Cronbach’s α for the reliability of negative affect measures was found to be 0.836.

#### 2.3.6 Psychological wellbeing scale—self-acceptance (PWB-SA)

Consisting of 14 items, the Self-Acceptance sub-scale of the Psychological WellBeing Scale developed by Ryff (1989) and adapted by Cenkseven and Akbaş (2007) was used for the current study. The CFA-based fit indices were as follows: *χ*2/df was 5.13, RMSEA was 0.080, CFI was 0.975, TLI was 0.957, and SRMR was 0.033. Given this, the model can be considered an excellent fit. Cronbach’s α for the reliability of the measurements was 0.852.

### 2.4 Data analysis

In the pilot study, exploratory factor analysis (EFA) was first applied to the data to see how the structure is in Turkish culture and to examine whether it differs from the structure of the original scale due to cultural reasons. EFA was conducted with SPSS (ver.25). Since the factors are related, Promax was chosen as the rotation method, and principal axis factoring was used for parameter estimation. Then, CFA was applied to the data collected in the pilot study.

To examine the scale’s construct validity, the CFA was executed with M*plus* (ver. 8.9) in both the pilot study and validation stages (Muthén and Muthén, 2017). Since some items did not show normal distribution, the mean adjusted maximum likelihood method (MLM) was used as a parameter estimation method. Generally speaking, the MLM (Satorra-Bentler’s Maximum Likelihood Mean Adjusted) is used to estimate the parameters with standard errors and a mean-adjusted chi-square test statistic. This approach is robust to non-normality (Muthén, 2009). The MLM chi-square test statistic is also known as the Satorra-Bentler chi-square. After the normality was verified, the correlation coefficients between the items were also examined; since these coefficients were lower than 0.90, it was determined that there was no multicollinearity (Field, 2009). With the CFA, the items’ factor loadings, significance, and error variances were analyzed. The model fit indices were examined by evaluating the *χ*^2^/df, Root Mean Squared Error Approximation (RMSEA), Comparative Fit Index (CFI), Tucker-Lewis Index (TLI), and Standardised Root Mean Square Residual (SRMR) values, and then interpreted according to the criteria values given in Table 1.

**Table 1:** LMS DFA fit indices and limits of acceptance.

Fit indices	Excellent fit	Acceptable/goodness of fit
*χ*^2^/df	*χ*^2^/df ≤ 3	*χ*^2^/df ≤ 5
RMSEA	≤0.05	≤0.08
CFI	0.95 ≤ CFI ≤ 1.00	0.90 ≤ CFI < 0.95
TLI	0.95 ≤ CFI ≤ 1.00	0.90 ≤ CFI < 0.95
SRMR	00 ≤ SRMR ≤0.05	0.05 ≤ SRMR ≤0.10

Baumgartner and Homburg (1996), Bentler (1980), Çokluk et al. (2012), Hu and Bentler (1999), Kline (2015).

To provide evidence for criterion-based validity in the validation phase, the relationship was examined between some variables, namely self-reported health status and self-defined humor, as well as the scores obtained with the LMS and those obtained from its sub-scales (NS, NP, and E). In addition, the relationship between the scores achieved through LMS and those obtained from MAAS, BFI-Openness, PANAS-PA, PANAS-NA, and PWB-SA scales was also examined. Spearman’s rank order correlation coefficients were estimated with the SPSS (ver. 25) program as some of the scores were found not to be normally distributed.

The last stage in the validation phase examined the measurement invariance according to gender. Measurement invariance was tested with multigroup CFA (MG-CFA), which consisted of configural invariance, metric invariance, scalar invariance, and strict invariance stages. Meredith (1993) suggests following a four-stage logical process and hypothesis testing methods to demonstrate measurement invariance: (1) Configural invariance: The fact that the factor structure of a psychological measurement tool is equal/invariant across groups means that it represents the same psychological construct across groups. (2) Metric invariance: It is used to determine whether different groups respond to the items similarly and whether the items’ regression slopes, also known as factor loadings, are equal/invariant between groups. (3) Scalar invariance: The constant number in the regression equations for the items is equal/invariant across groups, and invariance requires both metric invariance and equal origins in the measurement process. (4) Strict invariance: This stage determines whether or not the specific variances, in other words, the error terms of the items forming the measurement tool, are equal/invariant between the groups compared. When the next step was taken in the test of measurement invariance, the *Δ*𝐶𝐹𝐼 value, which expresses the change in CFI values in the previous stage and the new stage, was examined. It is recommended to examine the Δ𝐶𝐹𝐼 value in determining the differences between groups with the MG-CFA method (Cheung and Rensvold, 2002). In addition, Cheung and Rensvold (2002) stated that when the change of the CFI fit index value compared to the previous stage, i.e., the less restricted model, is in the range of −0.01 and 0.01, it will be possible to achieve measurement invariance. The MLM estimator was also used for the chi-square difference test. The significance of the difference between the hierarchical models was also tested by taking into account the scaling correction factor but not the chi-square values directly. A non-significant difference is considered evidence of invariance.

Finally, Cronbach’s *α*, McDonald’s *ω*, Composite Reliability Coefficient (CR), and average variance explained (AVE) were estimated for sub-dimensions to examine reliability. For the overall scale, stratified Cronbach’s α was taken instead of Cronbach’s α.

## 3 Results

### 3.1 Results of pilot study

A data set may fit with more than one CFA model, making it more appropriate to conduct an EFA first to account for possible cultural differences in the adaptation process. The data collected during the pilot study was initially analyzed using EFA to assess the construct’s relevance within Turkish culture. The suitability of the sample for factor analysis was tested using the Kaiser-Meyer-Olkin (KMO) measure and Bartlett’s Test of Sphericity. The KMO value was found to be 0.807, and Bartlett’s Test of Sphericity was significant (*p* < 0.001), indicating that the data were suitable for factor analysis. The EFA results revealed a three-factor structure, as confirmed by eigenvalues and scree plot examination, with these three factors explaining a total variance of 52.29%. The factor loadings of the items are presented in Table 2.

**Table 2:** Pilot study EFA result factor loadings.

	Novelty seeking (NS)	Novelty producing (NP)	Engagement (E)
I1	0.534		
I2		0.531	
I3		0.592	
I4			0.520
I5		0.064	
I6		0.726	
I7	0.554		
I8	0.761		
I9			0.902
I10	0.569		
I11		0.630	
I12			0.774
I13	0.613		
I14		0.660	

As seen in Table 2, all items except I5 were grouped under the factor identified in the original scale. It was observed that the fifth item did not have a factor loading above 0.30 in any of the factors (Hair et al., 2013). When the results of the CFA performed with the data collected from the pilot process were analyzed, the fit indices appeared to be as follows: *χ*^2^/df = 2.008, RMSEA = 0.053, CFI = 0.928, TLI = 0.911, and SRM*R* = 0.052. When these indices were compared with the criterion values, RMSEA, SRMR, CFI, and TLI provided evidence of acceptable/good fit, and the fit turned out to be excellent according to *χ*2/df. In general, it can be assumed that the data fit the model well. After the fit indices were examined, the factor loadings of each item, the significance of the *t* values of the observed variables, and the error variances were analyzed. Similar to the EFA results, except for the fifth item, the factor loadings of all items were higher than 0.30, the error variances were lower than 0.90, and the *t*-value was not significant. The fifth item, which was “I avoid thought-provoking conversations” in the source language, was adapted to the target language as “Düşünmeyi provoke eden konuşmalardan kaçınırım.” According to the feedback received through informal small talk during the pre-test phase, this item appeared to have a negative connotation for the students. Those in the English Language Teaching department emphasized this in particular. In cultural terms, provoking or provocation is used in the media and in everyday use in meanings that can be associated with offenses such as causing social protests and provoking other people. Therefore, the item in question was re-translated so that the final form of the scale in which the fifth item was changed could be used in the validation study. This translation used the term “teşvik eden-encouraging” instead of “provoke eden-provoking”.

### 3.2 Validation study

#### 3.2.1 Construct validity

In the validation study, the construct validity of the measures obtained from the scale was re-evaluated using CFA. The original scale comprises 14 items and 3 dimensions. CFA results indicated that variance between items 2 and 14, specifically “I generate few novel ideas” and “I am not an original thinker,” significantly contributed to the *χ*^2^ statistic and caused a decline in its value by 62.335. Upon theoretical and logical analysis, it was determined that these two items were similar in content and belonged to the same dimension, prompting the addition of an error covariance between items 2 and 14.

**Figure 1:**
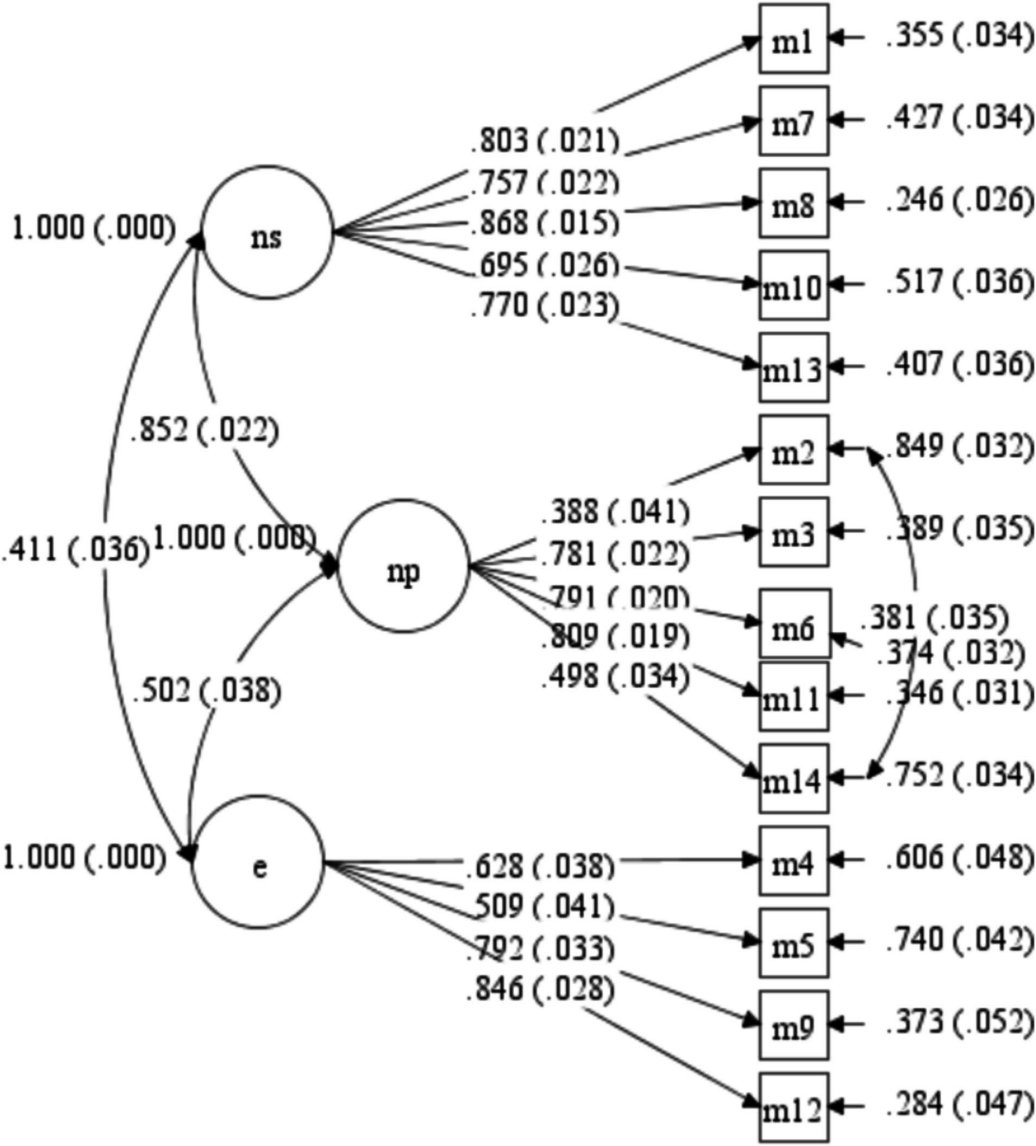
A path diagram of LMS.

Following this modification, the analysis yielded an RMSEA value of 0.080, indicating a good fit (Jöreskog and Sörbom, 1993). In addition, the CFI was 0.925, TLI was 0.906, and SRMR was 0.074, all suggesting that the model adequately fits the data (Hu and Bentler, 1999; Tabachnick et al., 2013). Another evaluated fit index, *χ*^2^/df, was found to be 3.964 after applying the correction factor. Overall, the fit statistics and factor loading values confirmed the structural integrity of the model. Figure 1 illustrates the path diagram, including factor loadings, significance levels, and error variances resulting from the CFA.

The path diagram in the Figure 1 shows all factor loadings are above 0.30. The factor loadings for the novelty-seeking dimension vary between 0.695 (I10) and 0.868 (I8), whereas those for novelty-producing vary between 0.388 (I2) and 0.809 (I11), and finally, they range from 0.509 (I5) to 0.846 (I12) for the dimension of engagement.

#### 3.2.2 Measurement invariance

Whether the measurement model was the same in different subgroups was examined for validation. The fact that the factor structures of a measurement tool are different in two groups provides information that a different psychological feature is measured in each group. In such a case, it is thought that the results obtained from two groups do not have the same outcome despite using the same measurement tool. The fact that a measurement model has the same structure in more than one group means that the factor loadings, correlations between factors, and error variances of the scale items in question are the same (Jöreskog and Sörbom, 1993). To this end, measurement invariance was examined according to the four-stage hierarchical model determined by Meredith (1993). Table 3 presents the fit indices for each model.

**Table 3:** The goodness of fit indices of the invariance in the model by gender.

	*χ* ^2^	df	*χ*^2^/df	RMSEA	SRMR	CFI	TLI
Configural invariance	430.749	146	2.950	0.089	0.079	0.915	0.894
Metric invariance	447.451	157	2.850	0.087	0.082	0.913	0.900
Scalar invariance	462.259	168	2.751	0.085	0.082	0.912	0.905
Strict invariance	469.986	185	2.540	0.079	0. 086	0.915	0.916

Table 3 shows the analysis of the difference between CFI values progressively among all models. If a comment is to be made based on the fit index values and *Δ*𝐶𝐹𝐼 value, it can be inferred that the model provides all invariances since each of them is <0.01 (Cheung and Rensvold, 2002). However, the 𝜒^2^ variance test between the two models should also be examined. The results of the 𝜒^2^ variance test between the two hierarchical models are given in Table 4.

**Table 4:** 𝜒2 variance test between hierarchical models.

Model	Δ𝜒2	Δ𝑑𝑓	*p*
Metric–configural invariance	12.506	11	0.327
Scalar–metric invariance	13.997	11	0.233
Strict–scalar invariance	18,390	17	0.365

As shown in Table 4, the analysis of variances in 𝜒^2^ values indicates that differences between the hierarchical models are not significant at 𝑝 < 0.01 level. Moreover, Δ𝜒^2^ value should be insignificant to indicate that the model and data fit are ensured. The results also show that all invariance models are included.

### 3.3 Results of reliability

As presented in Table 5, Cronbach’s *α* reliability coefficients for NS, NP, and E are 0.881, 0.807, and 0.778, respectively. The scores are considered to be reliable in terms of internal consistency. In scales consisting of more than one dimension, it seems more accurate to consider the stratified Cronbach’s α value for the reliability of the scores obtained from the whole scale (Tan, 2014). This value was 0.916 for the LMS overall scale. Another coefficient recommended for reliability estimation in measurement constructs is omega (*ω*) (McDonald, 1999). McDonald’s ω coefficients for NS, NP, and E dimensions and total score were 0.886, 0.797, 0.800, and 0.937, respectively.

**Table 5:** Reliability coefficients for LMS and sub-factors.

	CR	AVE	Cronbach’s α	McDonald’s ω	Stratified α
NS	0.886	0.609	0.881	0.886	–
NP	0.798	0.458	0.807	0.797	–
E	0.800	0.509	0.778	0.800	–
LMS	0.937	0.527	–	0.937	0.916

Besides finding the Cronbach’s α value for construct reliability, combined reliability (CR) values were also estimated. For convergent validity, it is recommended to find the AVE value of the average variance explained and determine the factor loading values with CFA (Hair et al., 2013). Cronbach’s alpha and CR values are expected to be ≥0.70, while the AVE value is expected to be ≥0.50 (Fornell and Larcker, 1981). As presented in Table 5, all of the scale’s CR, alpha, and omega reliability coefficients and sub-dimensions were above 0.798. Further, it can be suggested that the measurements are reliable regarding all coefficients. The average variance extracted values are higher than 0.50, except for NP. As Fornell and Larcker (1981) stated, the average variance explained may be a more conservative estimate of the validity of the measurement model, and “based on p_n_ (composite reliability) alone, the researcher may conclude that the convergent validity of the construct is adequate, even though more than 50% of the variance is due to error” (p. 46). As the composite reliability of the three factors is above the recommended level, the internal reliability of the measurement items is acceptable. Apart from proof of reliability, such a result can also be considered a proof of construct validity (Fornell and Larcker, 1981). From this standpoint, it seems clear that convergent validity was achieved.

The items of the Turkish version of the scale, which was finalized by considering the pilot study, construct validity and reliability results together, and the factor loadings of these items are given in Table 6.

**Table 6:** LMS Turkish Final Version Items and Factor Loadings.

Items	Novelty seeking	Novelty producing	Engagement
1. Bir şeyleri araştırmaktan hoşlanırım.	0.803		
2. Az sayıda özgün fikir üretirim.		0.388	
3. Birçok özgün katkıda bulunurum.		0.781	
4. İnsanların ne yaptıklarını nadiren fark ederim.			0.628
5. Düşünmeyi teşvik eden konuşmalardan kaçınırım			0.509
6. Çok yaratıcıyımdır.		0.791	
7. Çok meraklıyımdır.	0.757		
8. Bir şeyleri yapmanın yeni yollarını düşünmeye çalışırım.	0.868		
9. Değişimlerin nadiren farkında olurum.			0.792
10. Zihinsel olarak beni zorlayan etkinliklerden hoşlanırım.	0.695		
11. Yeni ve etkili fikirler üretmek benim için kolaydır.		0.809	
12. Yeni gelişmeleri nadiren fark ederim.			0.846
13. Bir şeylerin nasıl çalıştığını çözmeyi severim.	0.770		
14. Orijinal fikirleri olan biri değilimdir.		0.498	

### 3.4 Convergent and discriminant validity

After examining the construct validity and measurement invariance of the scale, the correlation coefficients between the scale and other variables considered to be related were estimated and given in Table 7.

**Table 7:** Correlation coefficients LMS, sub-factors, and criterion scores.

	Health	Humor	PA	NA	Openness	PWB-SA	NS	NP	E	LMS	MAAS
Health	–										
Humor	0.096*	–									
PA	0.149*	0.249*	–								
NA	−0.316*	−0.098*	−0.226*	–							
Openness	0.036	0.249*	0.415*	−0.184*	–						
PWB-SA	0.281*	0.150*	0.418*	−0.539*	0.269*	–					
NS	0.067	0.116*	0.366*	−0.088*	0.497*	0.241*	–				
NP	0.065	0.334*	0.410*	−0.172*	0.630*	0.296*	0.599*	–			
E	0.032	0.150*	0.125*	−0.162*	0.376*	0.158*	0.166*	0.378*	–		
LMS	0.072	0.259*	0.394*	−0.182*	0.654*	0.303*	0.779*	0.860*	0.660*	–	
MAAS	0.128*	0.037	0.204*	−0.305*	0.179*	0.367*	0.179*	0.213*	0.187*	0.252*	-

*Correlation is significant at the 0.01 level (2-tailed).

As seen in Table 7, the correlations between university students’ self-reported health and LMS, along with sub-factor scores, are not significant. However, the correlations between students’ self-defined humor, LMS scores, and sub-factor scores are significant. The LMS has a positive correlation with a positive effect (*r* = 0.394, *p* < 0.01) and a negative and significant correlation with a negative effect (*r* = −0.182, *p* < 0.01). Similar correlation coefficients were obtained for the sub-factors of the scale. The LMS scores were found to correlate significantly with the Openness to Experience scores, *r* = 0.654 (*p* < 0.01). The subscales’ correlations with Openness to Experience ranged from 0.376 to 0.630 (Novelty Seeking: 0.497, Novelty Producing: 0.630, Engagement: 0.376). The relationship between LMS and PWB-SA was analyzed to provide evidence for convergent validity, and it was found that the relationship was *r* = 0.303 (*p* < 0.01). Similarly, the relationship between the LMS sub-factors was also found significant. Further, a relationship was found between the LMS and MAAS, which measures the mindfulness construct from a different perspective and is considered evidence of convergent validity. The results showed a significant correlation between meditative and socio-cognitive mindfulness scores (*r* = 0.252, *p* < 0.01).

### 3.5 Correlation of latent factors

The analysis of the correlations between the factors indicates that there are moderate correlations between the sub-factors and that there is a high correlation with the total score. Table 8 shows the Spearman’s rank order correlation coefficients between variables.

**Table 8:** Correlation coefficients between sub-factors and overall scale.

	NS	NP	E	LMS
NS	–	0.644^**^	0.405^**^	0.854^**^
NP	0.644^**^	–	0.521^**^	0.882^**^
E	0.405^**^	0.521^**^	–	0.721^**^
LMS	0.854^**^	0.882^**^	0.721^**^	–

**Correlation is significant at the 0.01 level (2-tailed).

Table 8 shows that the correlations between the sub-factors are at a moderate level, and it is interpreted that the scores obtained from the factors can be summed. The final Turkish version of the LMS and a summary of the statistical values are presented in the Appendix.

## 4 Discussion

The LMS, rooted in Western perspectives, diverges from traditional Eastern mindfulness frameworks, emphasizing creativity and information processing, as noted by Haigh et al. (2011). Its adaptability across various cultures, evidenced by translations and validations in German, Persian, Japanese, and Portuguese, underscores the scale’s global applicability. Given the absence of a comparable scale in Türkiye that aligns with Langer’s educational focus, the adaptation of the LMS into Turkish presents a significant opportunity for researchers and educators in the region. This effort would not only fill a gap in the literature but also enhance the understanding and application of mindfulness in educational settings within Türkiye. Although mindfulness is prominent in psychology and education, it is not limited to these fields. Research related to mindfulness can also be found in business environments, and many studies identify the psychological structures it is commonly associated with (Davenport and Pagnini, 2016; Junça Silva and Caetano, 2021; Moafian et al., 2017; Pirson et al., 2012; Sherretz, 2011). Based on this, it is evident that the Turkish adaptation of the scale will contribute to the literature in these fields. It is expected that LMS will be widely used in the fields of education and psychology to describe the mindfulness levels of individuals. In addition, it is a scale that will be frequently used to reveal how this construct is related to other psychological constructs in Turkish culture.

In summary, this scale is thought to be used in descriptive, correlational, and experimental studies in social and clinical fields. Also, this adaptation, by ensuring that the LMS is culturally compatible and linguistically accessible, could facilitate further research and practice, benefiting educators, psychology researchers, and learners. Collaborations with local scholars and practitioners could also help use the scale to reflect cultural nuances, thus fostering a deeper engagement with mindfulness concepts in the Türkiye.

In the present study, LMS-14 was adapted to Turkish culture, after which its measurement invariance by gender was examined. The LMS has been adapted to many cultures in many studies before, which have provided evidence that valid and reliable measurements have been achieved. Some adaptations of the scale have been made on a 21-item form and some on a 14-item form. In our study, the factor structure of LMS-14 was analyzed with CFA to provide evidence for validity. As a result of CFA, the model-data fit seemed to be at a good level in such a way as to confirm the three-factor structure of the scale, namely, novelty-seeking, novelty-producing, and engagement, as in the original scale. As in the original scale structure, there are five items in the novelty-seeking factor, five in the novelty-producing factor, and four in the engagement factor in the Turkish version. Similar to this study, Junça Silva and Caetano (2021) also examined the LMS-14 factor structure in three different samples in the study adapted to Portuguese culture, revealing that the 14-item and 3-factor structure was confirmed in each sample. Besides that, Pagnini et al. (2018) also adapted the LMS-14 to Italian culture. As a result of their study, the socio-cognitive mindfulness structure consisting of authenticity-seeking, authenticity-generation, and commitment dimensions in accordance with the original scale structure was also confirmed in the Italian version of the scale. The Italian version’s fit indices seem similar to those of the Turkish version. Having been reported in a way consistent with these studies, the LMS-14, adapted to Turkish culture, had a 3-fact‑or and 14-item structure consisting of the same dimensions and items as the original version. Another adaptation of the 14-item short version of the scale was made to Japanese culture (Yang et al., 2023). In adapting the scale to Japanese culture, the original three-factor structure was found to be confirmed according to the model-data fit indices. Finally, the 14-item version of the scale was also adapted to French culture (Cook et al., 2023). The fact that the structure of the scale developed in the United States was found to be valid in Portuguese, Italian, Japanese, and French languages, as well as in Turkish culture, is a cross-cultural proof of the validity of the construct. In addition, the 21-item long form of the LMS appears to have been adapted to different cultures in the literature: Brazilian Portuguese, German, Malaysian, and Persian (Fernandes et al., 2023; Haller, 2015; Leong and Rasli, 2013; Moafian et al., 2017). It can, therefore, be considered that the cultures and languages in which both LMS-14 and LMS-21 were adapted are rich, and the CFA results of the scale provide evidence of validity in these cultures.

In this study, the correlations between various variables and the scores obtained from the LMS-14 and its sub-factors were examined to provide evidence for the convergent and discriminant validity of the measurements. For this purpose, the correlation between the scores and self-reported health was analyzed, yet no significant relationship was found between them. Such results are consistent with previous studies (Pirson et al., 2012; Pirson et al., 2018). In contrast to these results, Pagnini et al. (2018) found significant correlations between general and physical health and LMS and its sub-factors; Moafian et al. (2017) reported significant correlations between physical health and LMS and its sub-factors. However, although these relationships were statistically significant, they were found to be weak. Self-defined humor is another variable whose relationships with LMS-14 and its sub-factors were examined. In this study, the relationships between LMS-14, novelty-seeking, and engagement with self-defined humor were found to be significant yet weak, while the relationship between self-defined humor and novelty-producing turned out to be significant and moderate. Similar to the results of this study, Pirson et al. (2012) also found weak and moderately significant correlations between LMS-14 with its sub-factors and self-defined humor. When analyzed, the correlations between positive affect and LMS-14 and its sub-factors revealed that all the relationships were statistically significant. However, the correlation between positive affect and LMS-14, novelty-seeking, and novelty-producing was moderate and positive, while the one with engagement was positive but weak. These correlation coefficients were quite close to the coefficients in the study that led to the development of the scale’s original form (Pirson et al., 2012). Likewise, Junça Silva and Caetano (2021) and Moafian et al. (2017) also found the correlation between positive affect and LMS-14 to be moderate and positive. On the other hand, the correlations between negative affect and LMS-14 and its sub-factors were also analyzed to determine that all of the relationships were weak and negative but statistically significant. Such study results seem similar to those reported in several other studies (Moafian et al., 2017; Junça Silva and Caetano, 2021; Pirson et al., 2012).

Another variable considered for convergent validation is openness to experience. The reason is that personality traits are expected to be highly likely to influence levels of mindfulness (Costa and McCrae, 1992). Therefore, individuals’ openness to experience factor scores should correlate with their mindfulness scores. In the study, the relationship between openness to experience and LMS and its sub-factors was found to be statistically significant and positive, which is similar to those reported in the relevant literature (Pirson et al., 2018; Yang et al., 2023). The study by Pirson et al. (2012), where the original scale was created, is also in line with these results. In the same sense, Carson and Langer (2006) emphasized that self-acceptance is a conscious decision made by individuals as they take responsibility for their own lives and realize that important decisions are under their control. By definition, mindfulness also includes self-acceptance. In other words, individuals can accept themselves unconditionally only when they look at the world and themselves with awareness. From this perspective, the relationship between self-acceptance and mindfulness was examined with the expectation of a positive relationship. As a result of the study, the correlations between self-acceptance, one of the sub-factors of psychological wellbeing, and LMS-14 and its sub-factors were found to be significant and positive, as expected. However, it was concluded that while the correlation with LMS-14 scores was moderate, the one with its sub-factors was weak. Eventually, the correlation between the scores from the MAAS and the LMS and its sub-factors was analyzed. All of the statistically significant correlations were found to be positive and weak. This result is consistent with the original study (Pirson et al., 2012). Given the results of the other studies in the literature, the relationships between MAAS and LMS-14 scores also revealed similar correlations with respect to the results of this study (Fernandes et al., 2023; Junça Silva and Caetano, 2021; Yang et al., 2023).

To provide evidence for validity in the study, whether or not the measurements vary according to gender was analyzed according to the four-stage hierarchical model (Meredith, 1993). The analysis of the measurement invariance results led us to conclude that all of the configural invariance, scalar invariance, metric invariance, and strict invariance models showed model data fit. Since it shows model-data fit, including strict invariance, it can be assumed that there is intergroup invariance. As a result, it could be stated that the measurement process with the Turkish version of the scale does not differ by gender and measures the same factor structure. Finally, CR, AVE, Cronbach’s *α*, Stratified α, and McDonald’s *ω* were estimated to examine the reliability of the measurements. All of the values obtained are evidence of reliability according to the criterion values for both the overall scale and the measurements obtained with the sub-factors of the scale (Fornell and Larcker, 1981; Nunnally, 1978). Based on the results of the study, it was concluded that the 3-factor and 14-item structure was confirmed in the Turkish version of LMS-14 as in the original scale (Pirson et al., 2012), that convergent and discriminant validity evidence was consistent with the literature, and that reliable measurements were achieved. In general, the Turkish form of LMS-14 is thought to come to the forefront as it is a scale adapted to Turkish culture to measure socio-cognitive mindfulness.

Similarly, valid and reliable measurements have been made with this scale. A limitation of the study is that the validity and reliability studies of the scale were conducted for pre-service teachers. To expand the usage areas of LMS-14 in Türkiye, validation studies in different samples, such as business life, adults, high school students, and so on, can be recommended for future studies.

## Data Availability

The original contributions presented in the study are included in the article/Supplementary material, further inquiries can be directed to the corresponding author.
