# Supplementary material for: Validation and measurement invariance of the Langer mindfulness scale: the Turkish version
Source: Front Psychol. 2024 Nov 21;15:1474577. doi: 10.3389/fpsyg.2024.1474577 (PMC11619047; doi:10.3389/fpsyg.2024.1474577)
Supplement: Supplementary file 2 [file Presentation_1.pdf]

APPENDİX

LANGER BİLİNÇLİ FARKINDALIK ÖLÇEĞİ

Değerli katılımcı,

Aşağıda bilinçli farkındalığınızı ölçmeye yönelik ifadeler yer almaktadır. Bir ifadenin anlatımı ile ilgili karışıklık yaşarsanız, hiçbir fikriniz yoksa veya ne katılıyorum ne katılmıyorum diye düşünüyorsanız, "Kararsızım"ı işaretleyiniz. Yardımlarınız için teşekkür ederiz.

	**Kesinlikle Katılmıyorum**	**Katılmıyorum**	**Biraz Katılmıyorum**	**Kararsızım**	**Biraz Katılıyorum**	**Katılıyorum**	**Kesinlikle Katılıyorum**
	**1**	**2**	**3**	**4**	**5**	**6**	**7**
1. Bir şeyleri araştırmaktan hoşlanırım.							
2. Az sayıda özgün fikir üretirim.							
3. Birçok özgün katkıda bulunurum.							
4. İnsanların ne yaptıklarını nadiren fark ederim.							
5. Düşünmeyi teşvik eden konuşmalardan kaçınırım.							
6. Çok yaratıcıyımdır.							
7. Çok meraklıyımdır.							
8. Bir şeyleri yapmanın yeni yollarını düşünmeye çalışırım.							
9. Değişimlerin nadiren farkında olurum.							
10. Zihinsel olarak beni zorlayan etkinliklerden hoşlanırım.							
11. Yeni ve etkili fikirler üretmek benim için kolaydır.							
12. Yeni gelişmeleri nadiren fark ederim.							
13. Bir şeylerin nasıl çalıştığını çözmeyi severim.							
14. Orijinal fikirleri olan biri değilimdir.							

reverse coded items: 2, 4, 5, 9, 12, and 14.

SUMMARY STATISTICS

Fit Indices: RMSEA=0.08, CFI = 0.925, TLI = 0.906 and SRMR = 0.074.

Factor Loadings:

**Novelty Seeking**	
I1	0.803
I7	0.757
I8	0.868
I10	0.695
I13	0.770
**Novelty Producing**	
I2	0.388
I3	0.781
I6	0.791
I11	0.809
I14	0.498
**Engagement**	
I4	0.628
I5	0.509
I9	0.792
I12	0.846

Correlation between sub-factors:

	**NS**	**NP**	**E**	**LMS**
**NS**	-	.644**	.405**	.854**
**NP**	.644**	-	.521**	.882**
**E**	.405**	.521**	-	.721**
**LMS**	.854**	.882**	.721**	-

Reliability coefficients:

	**CR**	**AVE**	**Cronbach's α**	**McDonald's ω**	**Stratified α**
**NS**	0.886	0.609	0.881	0.886	-
**NP**	0.798	0.458	0.807	0.797	-
**E**	0.800	0.509	0.778	0.800	-
**LMS**	0.937	0.527	-	0.937	0.916
